# Differential diagnosis between psoriatic arthritis and hand osteoarthritis using indocyanine green-based fluorescence optical imaging

**DOI:** 10.3389/fmed.2025.1581265

**Published:** 2025-08-15

**Authors:** Benedict Drude, Øystein Maugesten, Stephanie G. Werner, Jens Klotsche, Gerd-Rüdiger Burmester, Gerhard Krönke, Marina Backhaus, Jörn Berger, Ida Kristin Haugen, Sarah Ohrndorf

**Affiliations:** ^1^Department of Rheumatology and Clinical Immunology, Charité-Universitätsmedizin Berlin, Berlin, Germany; ^2^Center for Treatment of Rheumatic and Musculoskeletal Diseases, Diakonhjemmet Hospital, Oslo, Norway; ^3^RHIO (Rheumatology, Immunology, Osteology) Duesseldorf, RHIO Research Institute, Düsseldorf, Germany; ^4^German Rheumatism Research Centre Berlin (DRFZ), Leibniz Association, Berlin, Germany; ^5^Institute for Social Medicine, Epidemiology and Health Economics, Charité – Universitätsmedizin Berlin, Berlin, Germany; ^6^Department of Rheumatology and Clinical Immunology, Parkklinik-Weißensee, Berlin, Germany; ^7^Xiralite GmbH, Berlin, Germany

**Keywords:** psoriatic arthritis, erosive hand osteoarthritis, fluorescence optical imaging, FOI, imaging, arthritis

## Abstract

**Introduction:**

Fluorescence optical imaging (FOI) visualizes enhanced microcirculation in the hands as a marker for inflammation. The correct diagnosis of psoriatic arthritis (PsA) and erosive hand osteoarthritis (EHOA) can be challenging. The aim of this study was to differentiate active PsA from EHOA using FOI.

**Methods:**

An atlas with FOI images of different grades of enhancement (FOIAS 0–3) and typical morphologic patterns (‘Streaky signals’, ‘Green/Blue Nail sign’, ‘Werner sign’, and ‘Bishop’s crozier sign’) for PsA and hand EHOA was created. Twenty FOI sequences of patients with PsA and EHOA were randomly mixed and scored by two blinded readers. All images were scored twice by one of the two readers. Inter- and intra-reader reliability for joint enhancement, morphologic patterns and diagnosis (PsA vs. EHOA) was calculated. Subsequently, one reader blinded to the diagnosis scored the remaining PsA (*n* = 54) and EHOA (*n* = 47) images using the same atlas.

**Results:**

Inter-reader reliability on joint enhancement was overall substantial (*κ* = 0.74), with substantial to almost perfect intra-reader reliability (*κ* = 0.88). Inter-reader reliability on all morphological patterns was fair (*κ* = 0.36) and substantial (*κ* = 0.68) in the intra-reader exercise with variation between the different patterns. Inter- (*κ* = 0.3) and intra-reader reliability (*κ* = 0.4) on diagnosis was fair. In analyses of all 101 cases (47 EHOA, 54 PsA), persons with EHOA presented significantly higher mean FOI sum scores in the PIP (38.98 vs. 20.00) and DIP joints (16.45 vs. 8.40) compared to the PsA patients. Regarding morphology, the ‘Werner sign’ was significantly more often detected in PsA than in EHOA (55.6% vs. 21.3%; *p* < 0.01).

**Conclusion:**

Joint enhancement in FOI can be reliably assessed using a predefined scoring method. The stronger enhancement in PIP and DIP joints for EHOA cases and the occurrence of ‘Werner sign’ in PsA cases may facilitate the differential diagnosis between the two diagnoses.

## 1 Introduction

Psoriatic arthritis (PsA) affects one in four patients with psoriasis (1) and presents heterogeneous clinical manifestations including joint inflammation, enthesitis, dactylitis, and axial disease, along with psoriatic skin and nail involvement (2). In the hands, wrist, metacarpophalangeal (MCP), proximal (PIP) and distal interphalangeal (DIP) joints can be affected by PsA. Inflammatory changes (synovitis, tenosynovitis/peritendinitis and enthesitis) in the peripheral joints combined with increased vascularity can early be detected by musculoskeletal ultrasound and also by magnetic resonance imaging (MRI) (3, 4). Inflammation in PsA can lead to erosive joint damage, which is particularly common in longstanding or more progressed disease (2).

Osteoarthritis commonly affects the hand joints. As one of three distinct hand OA phenotypes, erosive hand osteoarthritis (EHOA) primarily affects the DIP and PIP joints, and is associated with more severe inflammation, joint damage, pain and functional impairment in comparison with non-erosive hand OA (5, 6). Clinical symptoms also include swelling and redness in affected joint areas, as well as dysaesthesia, subluxation, instability and ankyloses (7). Established imaging modalities in clinical practice are conventional radiography, MRI and musculoskeletal ultrasound (8). Typical findings in radiography include narrowing of joint space, sawtooth and gull-wing erosions as well as osteophytes (7). Musculoskeletal ultrasound is more sensitive than conventional radiography, commonly displays synovial thickening and joint effusion and allows for the assessment of periarticular soft tissue (7). Higher power Doppler activity has also been described in patients with EHOA when compared to non-erosive hand OA or healthy individuals (9).

Another established diagnostic method that can aid in the differentiation between EHOA and PsA is synovial fluid (SF) analysis. While primarily used to identify crystal-induced and septic arthritis, SF analysis can also provide supportive information to distinguish degenerative from inflammatory joint diseases. In the context of EHOA and PsA, macroscopic SF characteristics—such as volume, color, and clarity—are particularly relevant. Compared to EHOA, PsA is typically associated with a lower SF clarity and a higher degree of joint effusion (10, 11). However, SF analysis is an invasive procedure with the potential risk of procedure-related complications including septic arthritis.

Fluorescence optical imaging (FOI) uses the fluorophore indocyanine green (ICG) to display enhanced microcirculation as a marker for inflammation in hand and finger joints (12–14). The strong affinity of ICG to plasma proteins and especially albumin aids this visualization of the vascular compartment (15). Several studies have shown great sensitivity of FOI in detecting early inflammation when compared to conventional MRI (12) or ultrasound (16) while displaying limited specificity. FOI is also able to detect subclinical synovitis (17), which has been associated with joint pain in hand OA patients (18). More recent research suggests that FOI might detect patients with skin psoriasis who are at risk for developing PsA (19). The FOI examination is both relatively quick when compared to standard ultrasound protocol for the assessment of hand joints and a safe diagnostic tool, as ICG is a long-established diagnostic agent with rarely occurring adverse effects (20).

In rheumatologic practice, differential diagnosis between PsA and EHOA can be challenging since both diseases may demonstrate similar patterns of distribution. Early and correct diagnosis of PsA and EHOA and their differentiation from each other are essential for further treatment decision. Using the high sensitivity attributed to FOI may be helpful in the differential diagnostic process. Previous research has pointed to specific joint morphologic patterns in FOI when comparing osteoarthritis with rheumatoid arthritis (RA) (21). Different morphologic patterns have been described in FOI that appear to be specific for PsA (22, 23).

Defined scoring methods for FOI have been validated for inter-reader reliability (24). Based on the “Berlin Method,” the Fluorescence Optical Imaging Score (FOIAS) (10) is a diagnostic tool for reading and scoring (0–3 grade of enhancement) FOI imaging sequences of both hands.

The aim of this analysis was to differentiate clinically active PsA from EHOA with FOI utilizing a standardized scoring method (FOIAS) while looking at joint signals and typical, predefined morphologic patterns.

## 2 Materials and methods

### 2.1 Study cohorts

FOI-sequences of PsA cases were obtained from data collected in the German-wide multicentric observational study ‘Optical Evaluation of Rheumatoid Arthritis’ (OPERA) in which FOI examinations of approximately 3,000 patients with a variety of rheumatologic hand joint disorders were screened for disease-related image patterns and compared to established imaging tools (25). The EHOA cases were selected from the longitudinal Nor-Hand study, which is a hospital-based cohort of 300 persons with hand OA between the age of 40–70 years. Persons with diagnosis of inflammatory arthritis as well as psoriasis were excluded from the Nor-Hand study (26).

In this analysis, we included persons with PsA from the OPERA study and persons with EHOA from the Nor-Hand study. Furthermore, at least one joint with synovitis grade ≥2 by ultrasound in greyscale (according to EULAR/OMERACT) or at least 2 swollen hand joints were required for inclusion. For the PsA cohort, we included persons with age ≤50 years to exclude concomitant hand OA.

### 2.2 FOI activity score and imaging atlas

To establish a precise and consistent scoring method for standardized FOI reading, a group of clinicians experienced in FOI interpretation refined the established, observer based scoring method Fluorescence optical imaging activity score (FOIAS), which is based on examining five images in three distinct phases in the FOI sequence as well as a composite image (12, 14, 21). Utilizing the XiraView® rainbow pallet template, the phases are characterized by distribution and washing out of the fluorescent dye in the hand joints. Shape of enhancement is assessed as well as color, which intensity scaling (gain in XiraView) can be adjusted in the Prima vista composite image (PVM). Both hands are assessed and rated separately.

**Figure 1:**
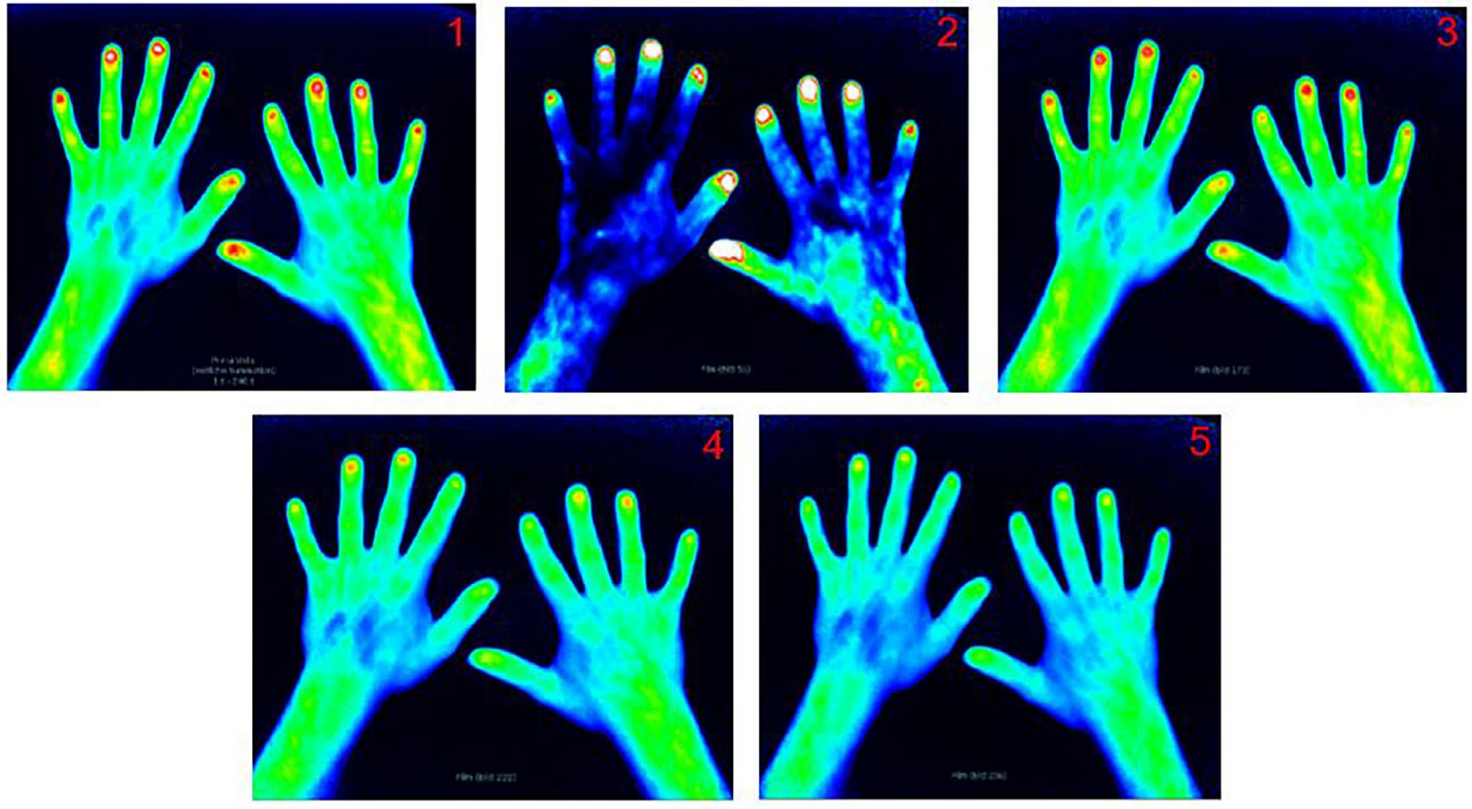
FOI image examples of the five different phases in FOIAS in a healthy control. Prima Vista mode (1), Phase 1 (2), Phase 2 first (3), Phase 2 middle image (4), and Phase 3 (5).

Phase 1 includes the period after application of the dye until it descends from distal fingertips to proximal. The last image before the dye (shown as yellow, red or white enhancement) descends is used for scoring. Phase 2 begins after Phase 1 ends and stops right before no more red enhancement is seen in the fingertips. For this phase, 2 different images are used for scoring. Phase 2 “first image” is defined as the first image in which no white enhancement can be detected in the fingertips. Phase 2 “middle image” is the image in the middle between Phase 2 “first image” and the first image of phase 3. Phase 3 begins in the absence of red enhancement in fingertips and lasts until the end of the examination. The first image of this phase is used for scoring. Finally, the Prima vista image (PVM) is a composite of the first 240 images. Example FOI images for a healthy control are displayed in Figure 1.

In these five images, joint signal enhancement is assessed on a semiquantitative 0–3 scale, where “0” corresponds to no red or white enhancement in the respective joint area. Grade 1 means yellow or red enhancement with red spots covering ≤50% of the enhanced joint area. Grade 2 signifies confluent strong red enhancement >50% of the enhanced joint area. White color spots may be present but do not cover >50% of the joint area. Finally, Grade 3 equates to confluent strong white color enhancement covering >50% of the joint area, with a surrounding red area possibly present.

Besides the semiquantitative scoring of joint signal enhancement, typical morphologic patterns for either EHOA or PsA were searched for in each of the FOI sequences. These patterns include the ‘streaky signal’ (21), a red signal separated by at least 2 stripes of yellow signal in the enhanced joint area, the ‘Green/Blue Nail Sign’ (22), referring to a sharply demarcated green area in projection of the nail that is surrounded by yellow or red color enhancement, the ‘Werner sign’ (14, 23), a triangular, arcuate enhancement from nail bed into the DIP area which can be described as being the shape of a reverse pyramid, and the ‘Bishop’s crozier Sign’ (27), corresponding to a shape like a question (or reverse question) mark next to the nail descending towards the DIP joint.

After agreeing on a suitable scoring method, an FOI atlas comprising of FOI images of healthy control subjects as well as example images for the different grades of enhancement for different hand joint groups (wrists, MCP, PIP, and DIP) was compiled. Additionally, example images of different morphologic patterns as mentioned above were added to the atlas. The atlas can be found in the Supplementary file 1.

### 2.3 FOI assessment

All patients received FOI examination using a Xiralite machine. The FOI examination included standardized intravenous application of 0.1 mg ICG per kilogram of body weight. The examinations were conducted under room temperature (approximately 20°C). FOI sequences were read using Xiraview Software Version 3.7.

All FOI sequences were rated by one reader (BD) according to the fluorescence optical imaging score (FOIAS). A total of 16 joints on each hand (wrist, thumb base, MCP1-5, (P)IP1-5, DIP2-5) were included in the analysis. The reader was blinded to diagnosis and had to determine whether the FOI sequence belonged to a patient with EHOA or PsA.

The four predefined patterns mentioned above were looked for in each case. Occurrence of these patterns was noted for each case on a binary yes/no (1/0) scale.

For validation, an inter-reader reliability exercise was performed. Two trained readers (BD and ØM) examined FOI sequences of 20 cases (*n* = 10 erosive hand OA and *n* = 10 PsA) that were not in the final analysis population. Both readers were blinded to diagnosis. They scored joint signal enhancement according to FOIAS and four morphologic patterns as mentioned above. Combining all FOI findings, each reader had to decide upon a diagnosis for each patient based on the FOI examination. Testing intra-reader reliability, one reader (BD) revisited the above-mentioned 20 cases in randomized order for a second time after 3 months and scored joint signal enhancement and morphologic patterns again as outlined above.

### 2.4 Statistical analysis

For statistical analysis, IBM SPSS Version 27.0 was utilized. Significance threshold was set to *p* < 0.05. Mean joint sum signals were added for both hands, calculated and compared between patients with PsA and erosive hand OA using *T*-test for independent samples. Morphologic pattern distribution in the two groups was compared by Pearson Chi-Square. To examine the ability to differentiate the two diagnoses (PsA vs. EHOA), the area under curve (AUC) was determined for each morphological pattern. Agreement on diagnosis and morphologic patterns for the inter- and intra-reader reliability test was assessed by Cohen’s kappa (*κ*). For the inter- and intra-reader reliability on scoring joint group signals, linear weighted Cohen’s kappa (*κ*) was utilized.

## 3 Results

### 3.1 Characteristics of OA and PsA cohorts

A total of 101 FOI-examinations were analyzed, 47 of those were patients with EHOA and 54 with PsA. Mean age (±SD) for the EHOA cohort was 64.0 ± 5.0 years, and for the PsA cohort it was 41.9 ± 8.0 years. The majority of patients were women (93.6% of EHOA patients and 68.5% of PsA patients). At the time of the FOI examination, 26 (48.1%) of PsA patients were treated with disease-modifying anti-rheumatic drugs (DMARDs); of those, 17 (65.3%) with conventional synthetic DMARDs and seven (27%) with biologic DMARDs. One patient with PsA received concomitant therapy with systemic corticosteroids and two PsA patients had documented nail involvement, (39 PsA patients with missing data). No serious adverse events were reported.

Median swollen joint count (SJC-66) was 7.0 (IQR 4–10) and 4.0 (IQR 3–8) in the EHOA patients and PsA patients, respectively, while tender joint count (TJC-68) was 8.0 (IQR 8–13) and 5.0 (IQR 2.3–9) in the EHOA patients and PsA patients, respectively.

The median CRP (mg/dl) was similar in persons with EHOA [0.23 (IQR 0.08–0.55)] and PsA [0.30 (IQR 0.07–0.8)] (Supplementary Table 1).

### 3.2 Joint group signal sum scores

In each FOI-Sequence, a total of 16 joints in each hand [wrist, thumb base, MCP1-5, (P)IP1-5 and DIP2-5] were evaluated, adding to a total of 3.232 assessed joints. Joint group signal sum scores can be found in Table 1. Total mean sum scores for PIP2-5 were significantly higher in persons with EHOA (39.0 ± 13.5) than in persons with PsA (20.0 ± 15.8). Persons with EHOA demonstrated higher values in the DIP joints in all phases except Phase 1. Wrists and IP joints also presented higher mean sum scores overall for EHOA, although not statistically significant. Total mean MCP joint sum scores were higher in persons with PsA (6.5 ± 14.4) than in persons with EHOA (3.1 ± 4.9), although not statistically significant (*p* = 0.29). Thumb base signals were rarely seen, and mean signals could only be computed for Phase 1, Phase 2 “first” and “overall.”

**Table 1:** Joint group mean sum scores in FOIAS (Fluorescence optical imaging score).

Joint group	Phase in FOI sequence	EHOA	PsA	*T*-test for independent samples
		Mean sum score (+SD)	Mean sum score (+SD)	*p*-value^b^
Wrist	PVM	0.49 (+0.72)	0.65 (+0.81)	0.29
	P1	0.06 (+0.32)	0.22 (+0.7)	0.13
	P2 first	2.68 (+1.37)	1.98 (+1.55)	0.01
	P2 middle	1.51 (+1.12)	1.22 (+1.23)	0.22
	P3	0.89 (+0.94)	0.68 (+1.06)	0.29
	Overall	5.64 (+3.52)	4.78 (+4.44)	0.29
Thumb base	PVM	–^a^	–^a^	–^a^
	P1	0.0	0.11 (+0.6)	0.18
	P2 first	0.28 (+0.72)	0.06 (+0.31)	0.05
	P2 middle	–^a^	–^a^	–^a^
	P3	–^a^	–^a^	–^a^
	Overall	0.28 (+0.72)	0.18 (+0.90)	0.53
MCP1-5	PVM	0.28 (+1.02)	1.00 (+2.08)	0.02
	P1	0.09 (+0.46)	1.26 (+3.73)	0.02
	P2 first	1.96 (+2.65)	2.15 (+4.36)	0.78
	P2 middle	0.51 (+1.02)	1.26 (+3.19)	0.12
	P3	0.21 (+0.66)	0.74 (+2.33)	0.13
	Overall	3.09 (+4.85)	6.46 (+14.35)	0.12
PIP2-5	PVM	8.94 (+2.82)	4.09 (+3.66)	<0.01
	P1	0.36 (+1.09)	0.63 (+1.7)	0.34
	P2 first	15.06 (+4.69)	7.96 (+5.9)	<0.01
	P2 middle	9.19 (+4.08)	4.54 (+4.55)	<0.01
	P3	5.26 (+3.73)	2.28 (+3.48)	<0.01
	Overall	38.98 (+13.52)	20.00 (+15.83)	<0.01
IP	PVM	0.64 (+0.82)	0.35 (+0.78)	0.07
	P1	0.36 (+0.85)	0.09 (+0.56)	0.06
	P2 first	1.49 (+0.91)	0.85 (+1.31)	<0.01
	P2 middle	0.51 (+0.75)	0.46 (+0.91)	0.76
	P3	0.11 (+0.31)	0.16 (+0.55)	0.55
	Overall	3.13 (+2.22)	2.00 (+3.59)	0.06
DIP2-5	PVM	4.32 (+2.7)	2.09 (+2.94)	<0.01
	P1	0.81 (+1.51)	0.39 (+0.92)	0.1
	P2 first	7.28 (+3.49)	3.60 (+4.02)	<0.01
	P2 middle	3.38 (+2.76)	1.58 (+2.52)	<0.01
	P3	0.66 (+1.72)	0.32 (+1.1)	0.25
	Overall	16.45 (+9.27)	8.40 (+9.31)	<0.01

a Not enough data to compute.

b Welch’s *T*-test.

### 3.3 Diagnosis and morphology

Reader diagnosis based on FOI findings matched clinical diagnosis in 78% (79/101) of all cases, showing moderate agreement with *κ* = 0.56 (*p* < 0.005, Area under curve = 0.78).

**Figure 2:**
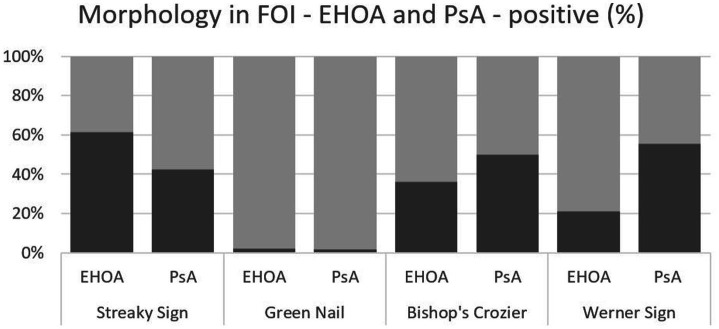
Overlapping of the four morphologic patterns in FOI—EHOA vs. PsA (dark gray bars) representing positive % for each pattern in relation to the total cohort (light gray bars).

‘Streaky sign’ was observed in 61.7% (29/47) of EHOA cases compared to 42.6% (23/54) of PsA cases (*p* = 0.06). ‘Green and Blue Nail signs’ were detected in one EHOA and in one PsA patient, respectively, and this difference was not statistically significant (*p* = 0.92). ‘Bishop’s crozier’ was found in 36.2% (17/47) of EHOA patients and in 50% (27/54) of PsA patients (*p* = 0.16). ‘Werner sign’ was seen in 21.3% (10/47) of EHOA cases and in 55.6% (30/54) of PsA cases (*p* < 0.005), as is presented by Table 2, Figure 2.

**Table 2:** Frequencies and differences of the four morphologies in EHOA vs. PsA.

Morphologic patterns	EHOA (columns %)	PsA (column %)	*p*-value	Area under curve
‘Streaky sign’	29 (61.7%)	23 (42.6%)	0.05	0.60
‘Green Nail sign’	1 (2.1%)	1 (1.9%)	0.92	0.50
‘Bishop‘s crozier’	17 (36.2%)	27 (50%)	0.16	0.57
‘Werner sign’	10 (21.3%)	30 (55.6%)	<0.01	0.67

### 3.4 Inter-reader reliability on joint groups

We found very good inter-reader reliability for (P)IP (*κ* = 0.81), wrists (*κ* = 0.69) and DIP joints (*κ* = 0.63), while the reliability for MCP joints was moderate (*κ* = 0.49). *Κ* values for all different joint groups are shown in Table 3. Looking at all joints, inter-reader reliability was substantial for PVM (*κ* = 0.73), Phase 2 “first” (*κ* = 0.75), Phase 2 “middle” (*κ* = 0.73) and Phase 3 (*κ* = 0.71), and moderate for Phase 1 (*κ* = 0.46).

**Table 3:** Inter- and intra-reader reliability: joint group signals in FOI.

Joint groups	Phase in FOI sequence	Inter-reader reliability	Intra-reader reliability
		Linear weighted kappa (*κ*)	Linear weighted kappa (*κ*)
Wrist	PVM	0.62	0.89
	P1	0.70	1.00
	P2 first	0.56	0.63
	P2 middle	0.55	0.67
	P3	0.69	0.62
	Overall	0.69	0.79
MCP1-5	PVM	0.36	0.77
	P1	0.73	0.87
	P2 first	0.49	0.70
	P2 middle	0.53	0.86
	P3	0.10	0.79
	Overall	0.49	0.78
(P)IP1-5	PVM	0.74	0.88
	P1	0.43	0.67
	P2 first	0.81	0.90
	P2 middle	0.76	0.90
	P3	0.77	0.83
	Overall	0.81	0.91
DIP2-5	PVM	0.59	0.76
	P1	0.16	–^a^
	P2 first	0.60	0.79
	P2 middle	0.64	0.86
	P3	0.62	0.88
	Overall	0.63	0.84

a Not enough values to calculate.

### 3.5 Intra-reader reliability on joint groups

Similarly, very good intra-reader reliability was found for (P)IP (*κ* = 0.91), DIP (*κ* = 0.84), wrists (*κ* = 0.79) and MCP joints (*κ* = 0.78) (Table 3).

### 3.6 Inter- and Intra-reader reliability on diagnosis and morphology

Regarding diagnosis, inter-reader reliability (*κ* = 0.30) and intra-reader reliability (*κ* = 0.40) were fair. In the inter-reader reliability exercise, we found moderate reliability for recognition of the ‘Werner sign’ (*κ* = 0.43) and fair reliability for ‘Bishop’s crozier’ (*κ* = 0.39) and ‘Streaky sign’ (*κ* = 0.27) (Supplementary Table 2). ‘Green or Blue Nail sign’ presented not often enough to compute *κ*. The Intra-reader exercise featured substantial reliability for ‘Werner sign’ (*κ* = 0.79) and moderate reliability for ‘Streaky sign’ (*κ* = 0.6) and ‘Bishop’s crozier’ (*κ* = 0.49). As in the inter-reader reliability exercise, ‘Green and Blue Nail sign’ were not observed often enough to produce dependable data.

## 4 Discussion

Correct diagnosis of EHOA and PsA and their differentiation from each other remains a clinical challenge but is essential for further treatment decision. The aim of this analysis was to differentiate active PsA from EHOA utilizing FOI and a standardized scoring method to evaluate joint signals and typical morphologic patterns, supported by a reference image atlas. The application of FOI in clinical use for this differentiation offers practical advantages, as the examination is carried out quickly and does not need a medical doctor present during the examination. Injection of ICG might be a constraint, although adverse events are only rarely observed (20).

### 4.1 Reliability and observer agreement

Pre-testing calibration and evaluation of inter- and intra-reader reliability was conducted before joint scoring and morphological assessment. We observed substantial inter-reader reliability for scoring wrists, PIP and DIP joints with phase 1 displaying the highest inter-reader reliability for wrists and MCP joints, while phase 2 performed better for PIP and DIP joints. These findings align with a previous study by Werner et al. (12), who demonstrated comparable inter-reader reliability for trained readers when applying FOIAS to patients with early arthritis. Another study by Maugesten et al. (24) noted similarly solid inter-reader reliability when comparing the “Berlin Method,” on which the FOIAS used in this study is mostly based, to other observer-based FOI scoring methods in patients with RA and hand OA. Both studies emphasize the need for pre-testing calibration, noting limited inter-reader reliability for inexperienced readers (12, 24). This was addressed by compiling the above-mentioned FOI atlas as a reference for this study and to aid in further training purposes. So far, these results reinforce the usefulness of FOIAS as a reliable tool to assess hand and finger joint affection in FOI.

Nevertheless, and despite these measures, the overall inter-reader agreement for morphological patterns was lower than expected, which highlights the inherent limitations of the observer-based character of FOIAS.

### 4.2 Differences in FOI signal intensity

We found significantly stronger enhancements in PIP and DIP joints of EHOA cases when compared to PsA. This observation my result from different influencing factors. Firstly, active disease was an inclusion criterion for both groups, however the EHOA group presented higher clinical disease activity (referenced by higher SJC and TJC scores), to which higher FOIAS can be attributed. Secondly, 48% of PsA patients underwent systemic therapy (including biologics) during FOI assessment, which could have reduced the strength of joint signal accumulation in this cohort. Ideally, imaging assessment should be conducted in treatment-naïve cohorts. This, however, appears difficult for PsA, as a relevant number of psoriasis patients undergo systemic treatment even before diagnosis of PsA (19, 28–30). Thirdly, the EHOA cohort was generally older than the PsA group (64 vs. 42 years) and predominantly composed of postmenopausal women, thus being at higher risk for more severe EHOA progression rendered in more pronounced FOI joint signals (31). Prior studies have observed generally mildly pronounced FOI joint signals in PsA (28), which further emphasizes the necessity of detecting extraarticular disease manifestations in this patient population. As expected, affection of the CMC joint was more pronounced in persons with EHOA than in persons with PsA. Previous research has pointed out the difficulty of visualizing inflammation by FOI in the thumb base, possibly due to a larger amount of surrounding soft tissue in the CMC joint than the PIP and DIP joints. As FOI has limited tissue penetration depth due to reduction of fluorescent signals by up to 10-fold per cm of depth (32), this might provide an explanation for the limitation of FOI in EHOA (18).

In our analysis of the patients with PsA, the most pronounced FOI signals and highest sum scores were observed in the PIP joints in phase 2 (first image). This is in line with previous research by Erdmann-Keding et al. (28), who also reported more frequent pathological findings for PIP joints in PsA patients in Phase 2 when comparing FOI to ultrasound. This further highlights the high sensitivity of signal detection attributed to Phase 2 of the FOI sequence (14).

### 4.3 Morphologic patterns

Among the morphologic patterns, the ‘Werner sign’ was the only feature that significantly differentiated PsA from EHOA, occurring more often in persons with PsA. While this pattern exhibited moderate inter- and intra-reader reliability and had a moderate AUC value of 0.67, it requires further validation to confirm its potential value in the differential diagnosis of PsA vs. EHOA. The hypothesis that the ‘Werner sign’ represents an (early) affection of the synovio-enthesial complex in PsA (14, 33) is of interest, but warrants further investigation. Comparative analysis of FOI sequences of PsA patients and other rheumatic hand diseases may provide additional insights.

The ‘streaky sign’ and ‘Bishop’s crozier’ showed fair to moderate inter- and intra-reader reliability. Previous research by Glimm et al. (21) has linked the occurrence of streaky signs in EHOA patients to degenerative joint damage, which could explain the more frequent, although not statistically significant presentation of this sign in our EHOA cohort. Further research should clear the role and pathophysiological correlate of this pattern. The ‘Bishop’s crozier’ did not differ in prevalence between persons with EHOA and PsA. This is consistent with prior findings from Wiemann et al. (22), who noted limited sensitivity and specificity of this extraarticular pattern in distinguishing PsA from RA (27).

In the current study, the ‘Green or Blue Nail signs’ were uncommon in both groups, suggesting no utility in differentiating PsA from EHOA. This is also in line with previous research noting limited sensitivity for this pattern (22). The low prevalence of these patterns might be attributed to the absence of documented psoriatic nail involvement, which has been suggested to be linked to this extraarticular feature (22).

Additional morphologic structures separated from joint signal enhancement may be useful with regards to differentiation of PsA and EHOA, as well as for other inflammatory diseases with affection of the hands. An earlier study by Schmidt et al. found higher occurrence of subdermal areal skin enhancement in FOI sequences of patients with psoriasis and PsA when compared to RA (34). In future studies, different and distinct morphological presentation could help to achieve a reliable differential diagnosis when screening for PsA. A combination of the Werner sign and above mentioned, subdermal skin enhancement might prove useful for this.

### 4.4 Potential of AI in pattern recognition

Advancements on Artificial intelligence (AI)-assisted pattern recognition may further elevate the diagnostic accuracy of FOI. A recent study by Rothe et al. (35) demonstrated that machine learning algorithms could effectively differentiate RA, OA and connective tissue diseases based on a small number of FOI features. The authors suggested a possible further reduction of used features at cost of relatively small performance loss. It must be argued, however, that overlap-syndromes are not accounted for in this current approach, as the authors also acknowledge. So far there have been, to our knowledge, no studies that apply such approaches to the differentiation of PsA and EHOA. Incorporating the ‚Werner sign‘and other disease-specific features into AI-driven models may refine differential diagnosis in future research.

### 4.5 Study limitations

This study has several limitations. Its retrospective design limits control over confounding variables, such as treatment status and disease duration. The EHOA cohort was, as acknowledged above, both older and clinically more active when compared to the PsA group, which may have contributed to stronger FOI joint signals. However, a relatively younger PsA cohort also lowers the risk for co-occurring EHOA in this group. Almost half of the PsA cohort had systemic treatment with DMARDs at the time of FOI examination, potentially diminishing FOI enhancement and confounding comparisons. Additionally, the lack of documentation of PsA disease duration and psoriatic nail involvement may have influenced the FOI interpretation, especially in regard to extraarticular manifestations in FOI as mentioned in the case of the ‘Green Nail’ phenomenon. PsA cases had documentation of DAS28, which, although often in clinical use (36) has been validated for RA and shown to be inferior to more established scores in PsA patients, such as the Psoriatic Arthritis Disease Activity Score (PASDAS) or the GRAppa Composite scorE (GRACE) (37). A prospective study design with age- and gender-matched cohorts would provide the opportunity to mitigate these biases.

## 5 Conclusion

To our knowledge, this is the first study to explore FOI for differentiating EHOA from PsA. As an initial step in this direction, our findings offer a preliminary basis for identifying potentially distinguishing patterns between these two conditions. We found that FOIAS proved to be a reliable tool for evaluating joint-related FOI signals, supporting its utility in structured assessments. While the ability of FOI to clearly differentiate between EHOA and PsA remains limited at this stage, certain pattern signals—such as strong activity in PIP and DIP joints—may be more suggestive of EHOA. FOI presents a safe, quick, and feasible adjunct to clinical examination, musculoskeletal ultrasound, and other conventional imaging procedures.

Future studies should build on these exploratory insights by refining the characterization of morphological patterns and further investigating extraarticular signal manifestation in FOI. For this endeavor, AI-assisted analyses could also be incorporated to improve diagnostic accuracy.

## Data Availability

The raw data supporting the conclusions of this article will be made available by the authors, without undue reservation.
