# Supplementary material for: Differential diagnosis between psoriatic arthritis and hand osteoarthritis using indocyanine green-based fluorescence optical imaging
Source: Front Med (Lausanne). 2025 Aug 15;12:1581265. doi: 10.3389/fmed.2025.1581265 (PMC12394491; doi:10.3389/fmed.2025.1581265)
Supplement: Supplementary file 1 [file Data_Sheet_1.docx]

# Supplementary material

**Supplementary table 1:** Demographic data on the two included cohorts: hand OA, hand osteoarthritis and PsA, psoriatic arthritis

|  | Hand OA, n = 47 | PsA, n = 54 |
| --- | --- | --- |
| Mean age (SD) | 64±5 years | 41.9±8,3 years |
| Sex (%total) | male: 3 (6.4%)  female: 44 (93.6%) | male: 17 (31.5%)  female: 37 (68.5%) |
| Median SJC^a^ (IQR) | 7 (4-10) | 4 (3-8) |
| Median TCJ^b^ (IQR) | 8 (8-13) | 5 (2.25-9) |
| Median CRP in mg/dl (IQR)^c^ | 0.23 (0.08 – 0.55) | 3 (0.65 – 8) |

Key: ^a^ 2 under basic therapy, 15 under methotrexate, 7 under biologicals; ^a^ swollen joint count; ^b^tender joint count; ^c^ 2 entries missing

**Supplementary table 2:** Inter- and intra-reader reliability on four morphologic patterns

| Morphologic pattern | Inter-reader reliability | Intra-reader reliability |
| --- | --- | --- |
|  | Kappa (Κ)^a^ | Kappa (Κ)^a^ |
| ‚Streaky sign‘ | 0.27 | 0.6 |
| ‚Green Nail sign‘ | -^b^ | -^b^ |
| ‚Bishop’s Crozier‘ | 0.38 | 0.48 |
| ‚Werner sign‘ | 0.42 | 0.79 |
| **All patterns combined** | 0.36 | 0.68 |

Key: ^a^using linear weighted Cohens Kappa; ^b^ not enough values to compute Κ

**Supplementary file 1:** FOI imaging atlas.docx

FOI imaging atlas with example images of healthy control, FOIAS demonstration in typical finger joint findings as well as example images of morphologic patterns.
