# Supplementary material for: Differential diagnosis between psoriatic arthritis and hand osteoarthritis using indocyanine green-based fluorescence optical imaging
Source: Front Med (Lausanne). 2025 Aug 15;12:1581265. doi: 10.3389/fmed.2025.1581265 (PMC12394491; doi:10.3389/fmed.2025.1581265)

**
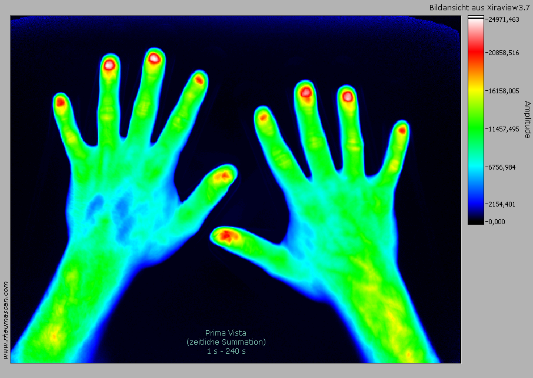
Updated Fluorescence Optical Imaging Atlas**

with special focus on Hand Osteoarthritis (OA)

and Psoriatic arthritis (PsA)

-
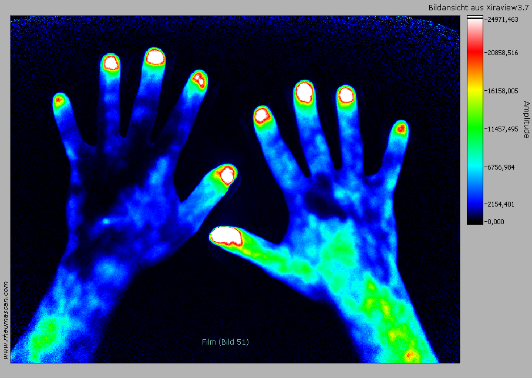
Definition of FOI activity score (FOIAS)
- Example images (of grade 1-3) in

DIP, PIP, MCP and wrist

- Suspected Hand OA and PsA features


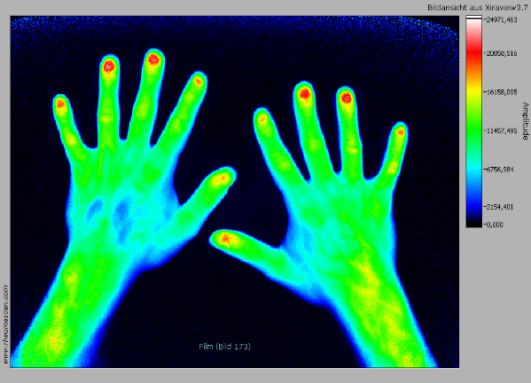


**FOI activity score (FOIAS)**^1,2^ **– Definition of phases**

Always in all cases, the film (0-360min.) should be watched.

One predefined image from phase 1, 2 and 3 and a composite image, Prima Vista mode (PVM), should be assessed per patient.
Phase 1-3 are defined based on the distribution and washing out of the fluorescent dye. Phases are analyzed in right and left hand separately.

Joint signals should be assessed for the shape of enhancement as well as the colour. Gain can be adjusted if the PVM image is without white enhancement in the fingertips or there is no red or white enhancement in joint areas. Gain is adjusted in PVM until red enhancement is seen in joint areas or fingertips. If applying a different XiraView® template than rainbow (i.e temperature, black & white), the corresponding definition for increased signal intensities should be used.

1. *Werner et al., Ann Rheum Dis. 2012 Apr; 71(4):504-510*
2. *Glimm et al., Ann Rheum Dis. 2016 Mar; 75(3):566-570*

**Definition of phases (using XiraView® rainbow template)**

- **Phase 1:** Includes the period after application of the dye until the dye descends from the fingertips from distal to proximal.
- Phase 1 image: The last image before the dye (shown as yellow, red or white enhancement – not green) descends from the fingertips from distal to proximal is used for scoring.

**Phase 2:** begins after the end of Phase 1 and stops right before no more red enhancement is seen in the fingertips.

- Phase 2 images: **First** is defined as the first image when no white enhancement can be detected in the fingertips. The **middle** image is the image in the middle between the first image of phase 2 and the first image of phase 3. Both first and middle image should be assessed.
- **Phase 3:** The beginning of phase 3 is characterized by absence of red enhancement in fingertips. Phase 3 lasts until the end of the examination.
- Phase 3 image: The first image of phase 3 should be assessed.
- **PVM:** Composite image defined by the XiraView® software based on the 240 first images.

**Example images of FOIAS phases 1, 2 (first image and middle), 3 and PVM in a healthy control:**


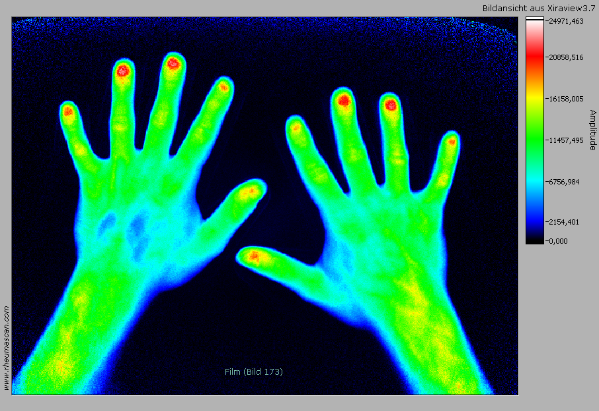

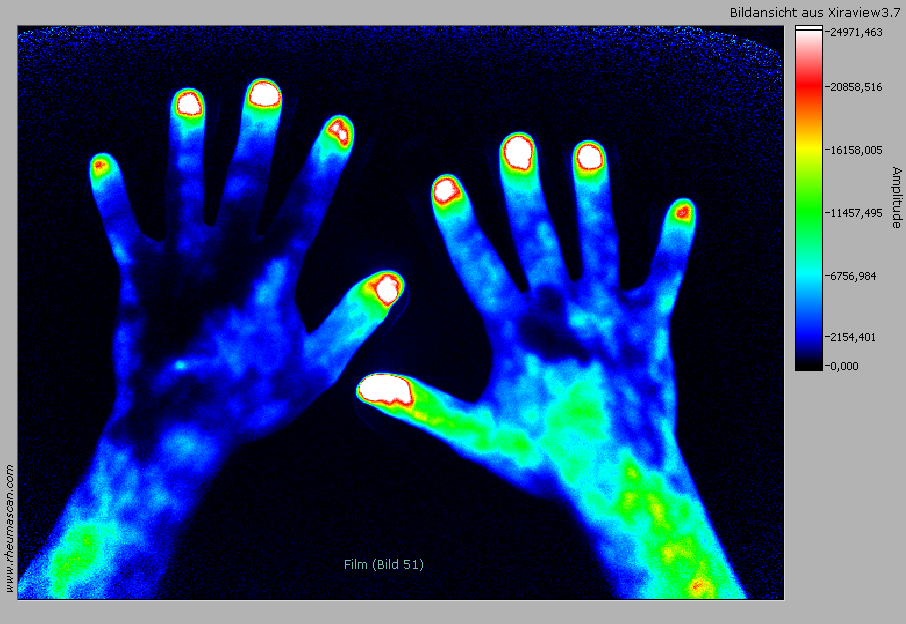

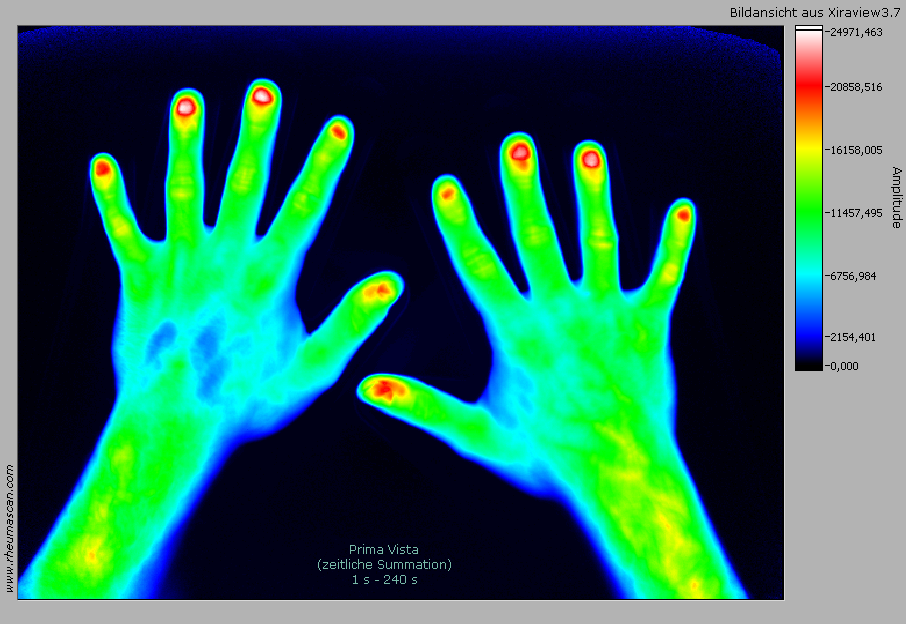


Phase 1

Phase 2 (first)

PVM


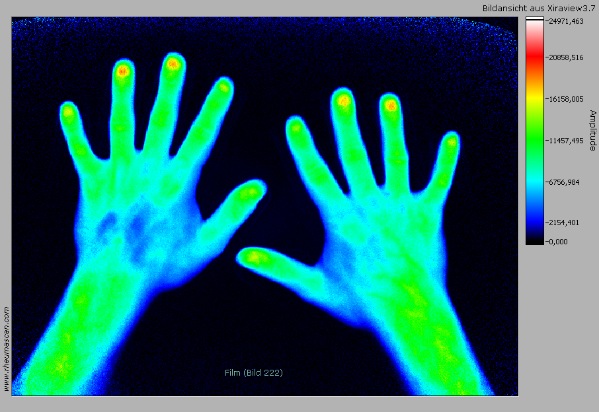

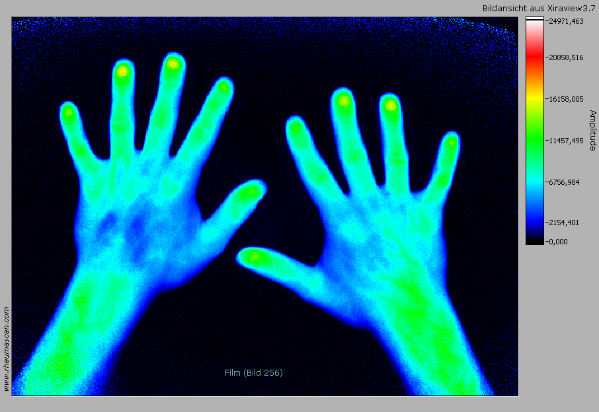


Phase 3

Phase 2 (middle)

**FOIAS – Definition of grading**^1^

The semiquantitative ‘Fluorescence Optical Imaging Activity Score (FOIAS)’ applies the rainbow pallet in the XiraView® software. The enhancement is evaluated based on the size and colour intensity of the enhanced joint area. In other templates, the corresponding definition for increased signal intensities should be used.

- **Normal:** No red or white enhancement
- **Grade 1:** Yellow and red enhancement of the joint area with red spots covering ≤50% of the enhanced joint area.
- **Grade 2:** Confluent strong red enhancement >50 % of the enhanced joint area. ≤50 % white spots may be present.
- **Grade 3:** Confluent strong white enhancement >50% of the enhanced joint area. Red surrounding area may be present.

Exception 1: Few, irregular, non-confluent pixel-sized red dots are not graded as enhancement. See examples on page 5.

Exception 2: If the enhancement has the same shape as a vessel, the colour/shape of the vessel is not included in the grading. The surrounding area can still be graded if there is enhancement outside of the vessel. See examples on page 6.

1. *Werner et al., Ann Rheum Dis. 2012 Apr; 71(4):504-510*

**Exception 1:**
Non-confluent pixel-sized red dots without graduation for FOIAS.


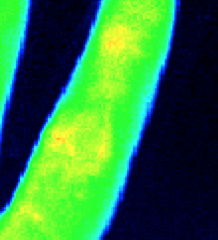

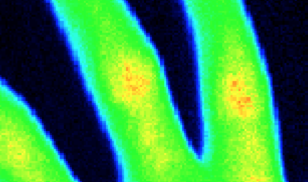

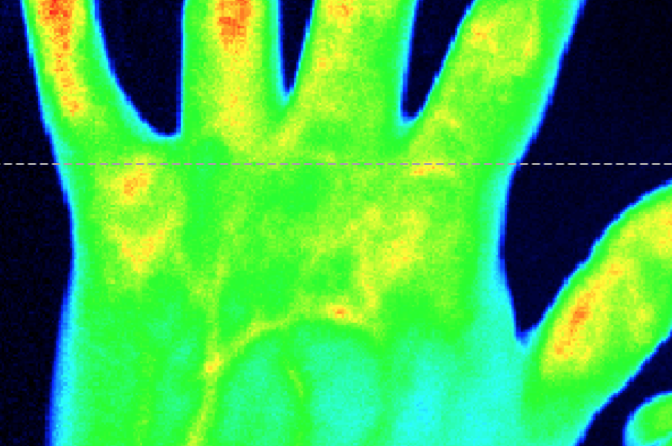

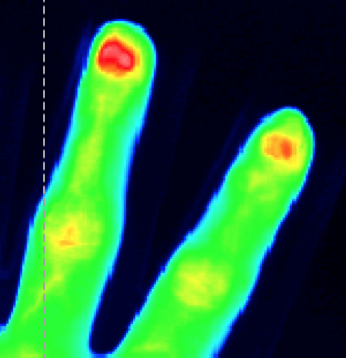


**Exception 2:**

Vessels


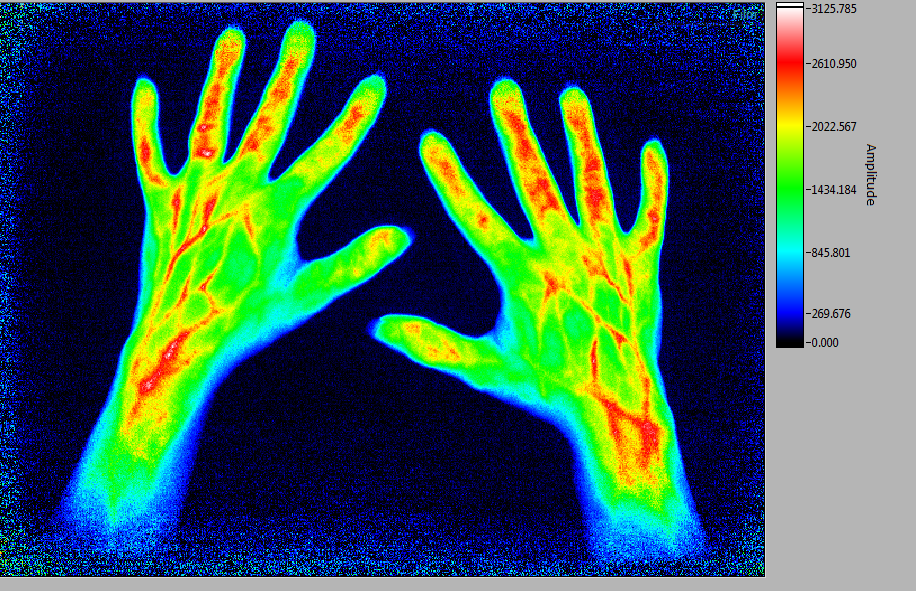

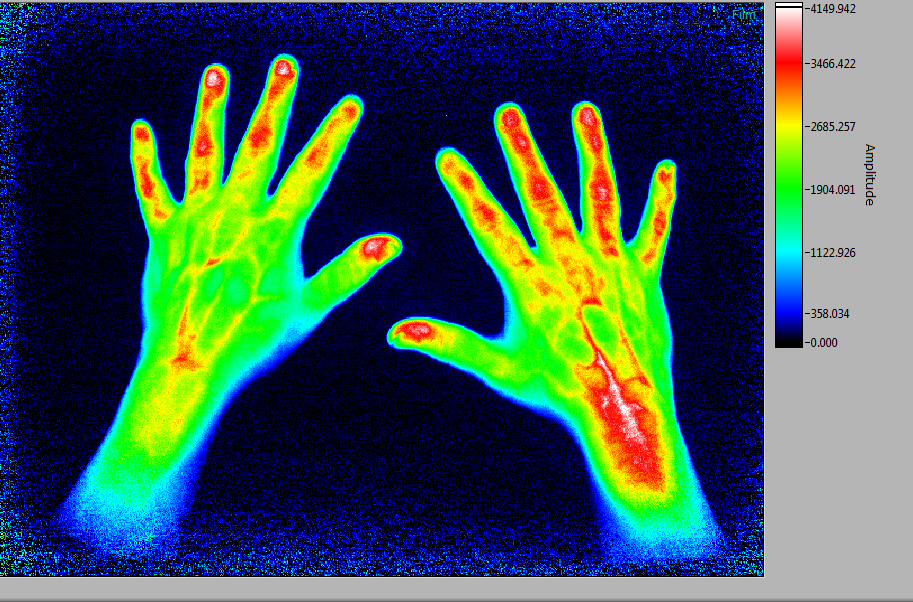

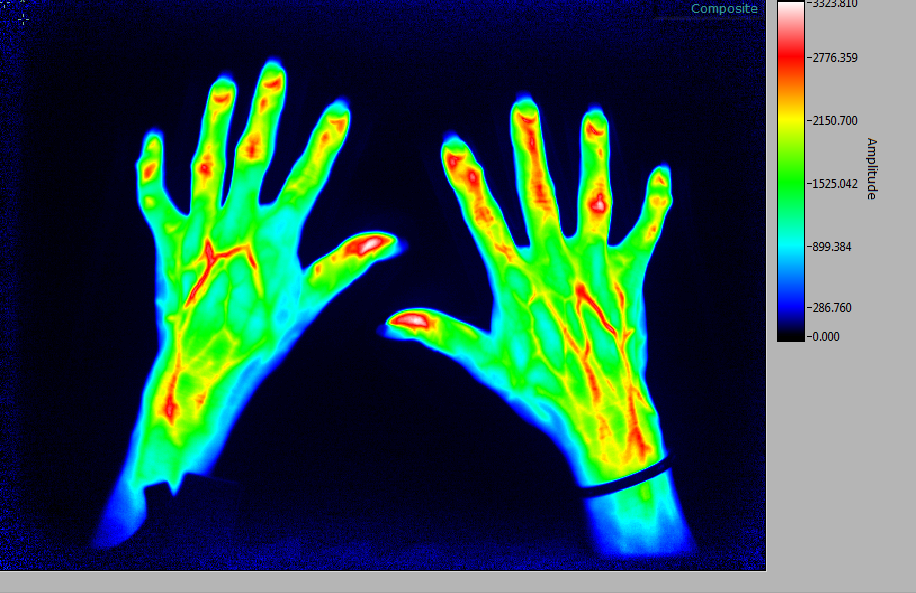


**Atlas of grade 1-3 in different joint groups**

Distal interphalangeal joints (DIP)


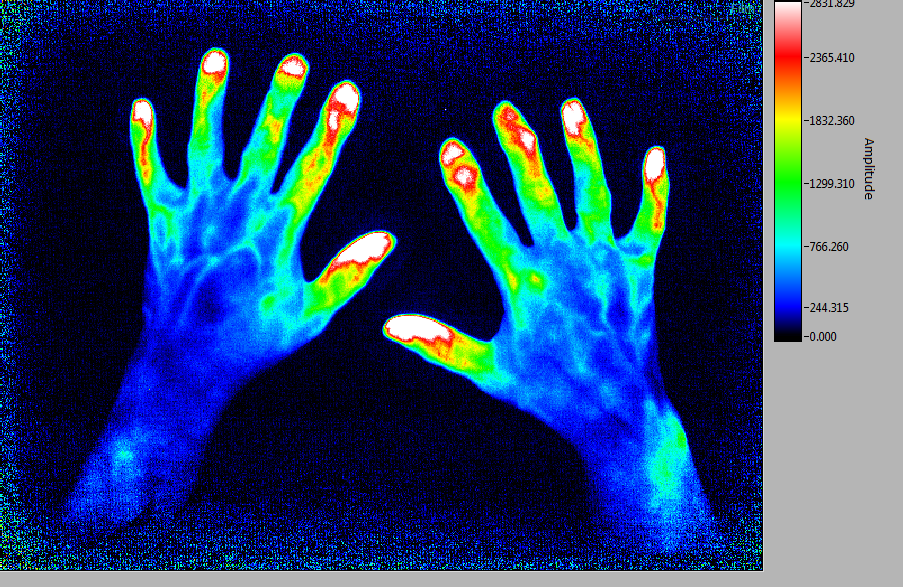

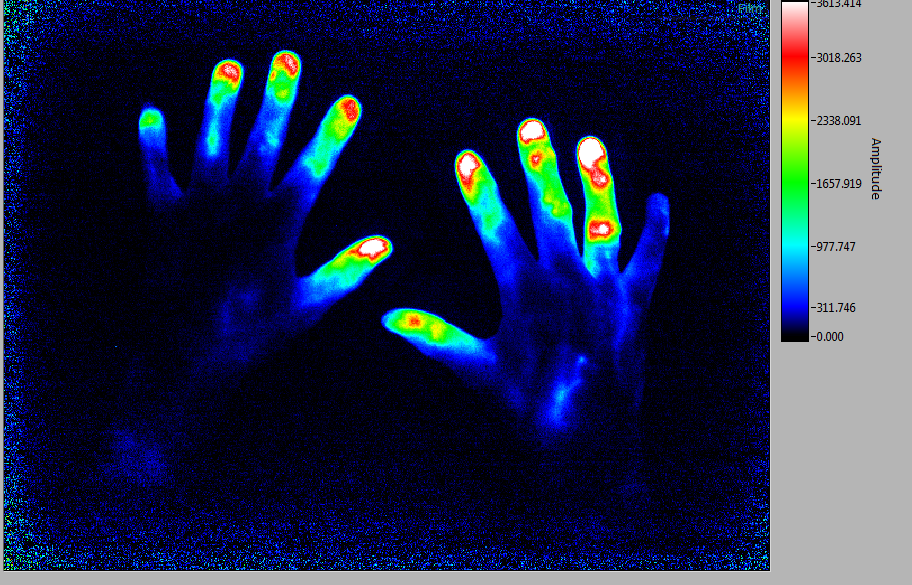

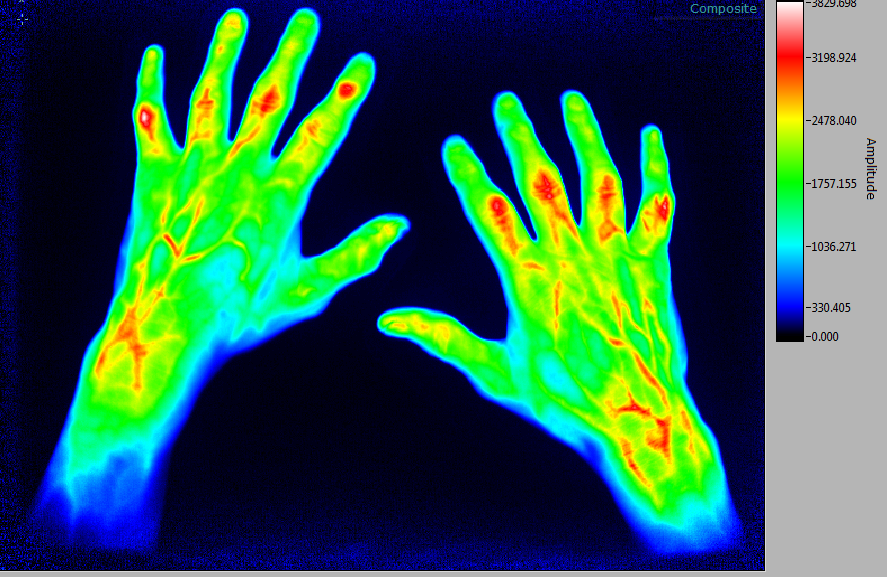


Grade 1 Grade 2 Grade 3

Proximal interphalangeal joints (PIP)

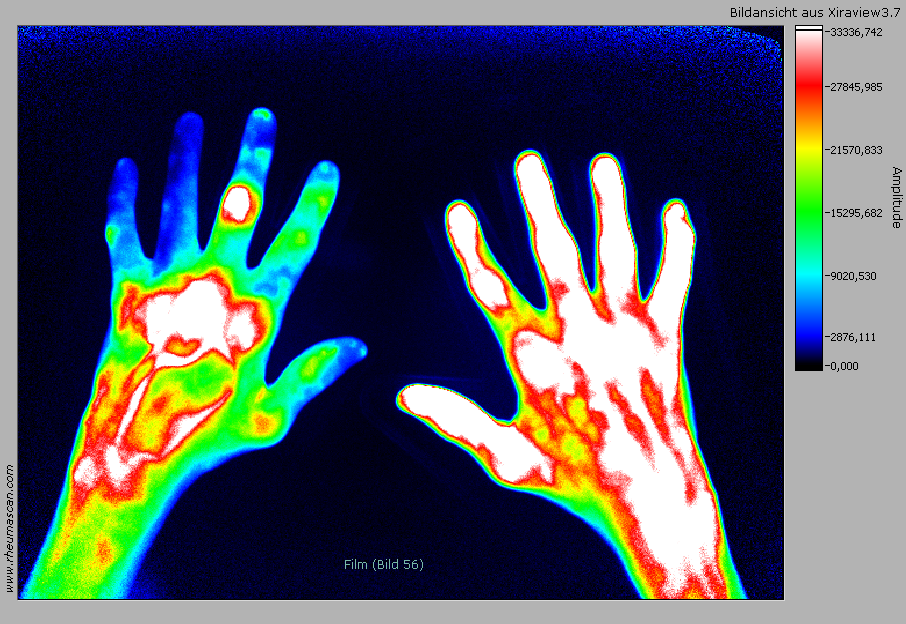


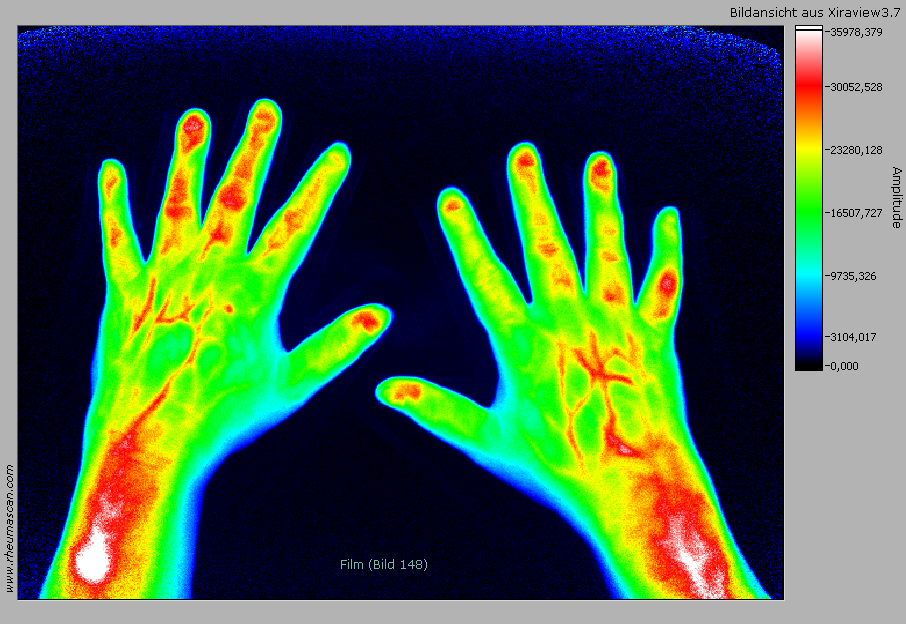

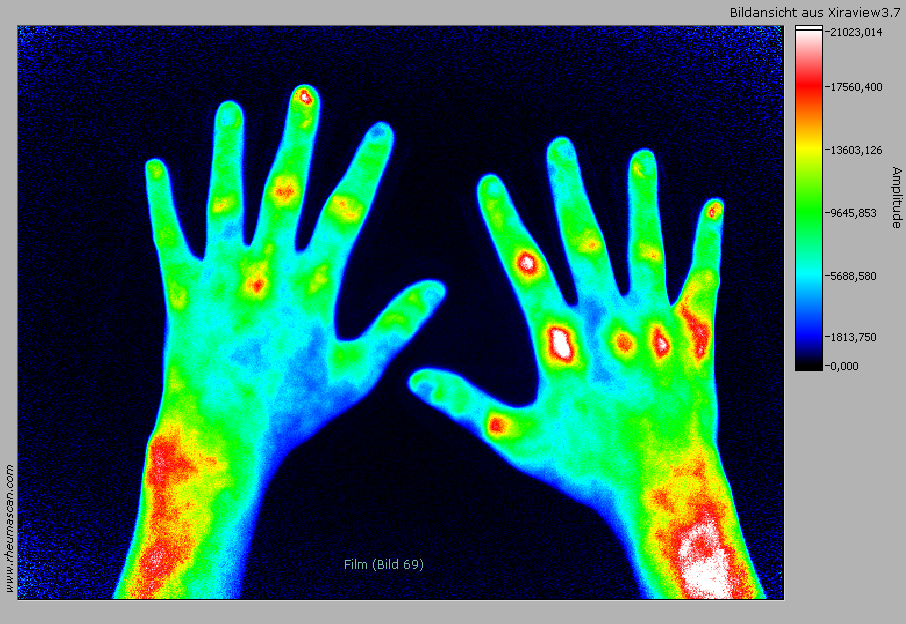


Grade 3

Grade 1 Grade 2

Metacarpophalangeal joints (MCP)


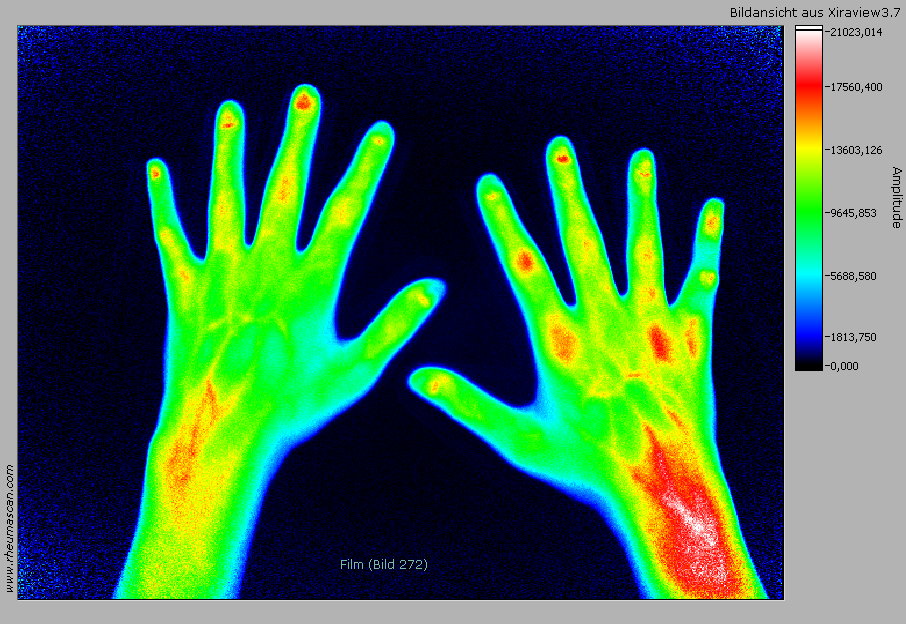


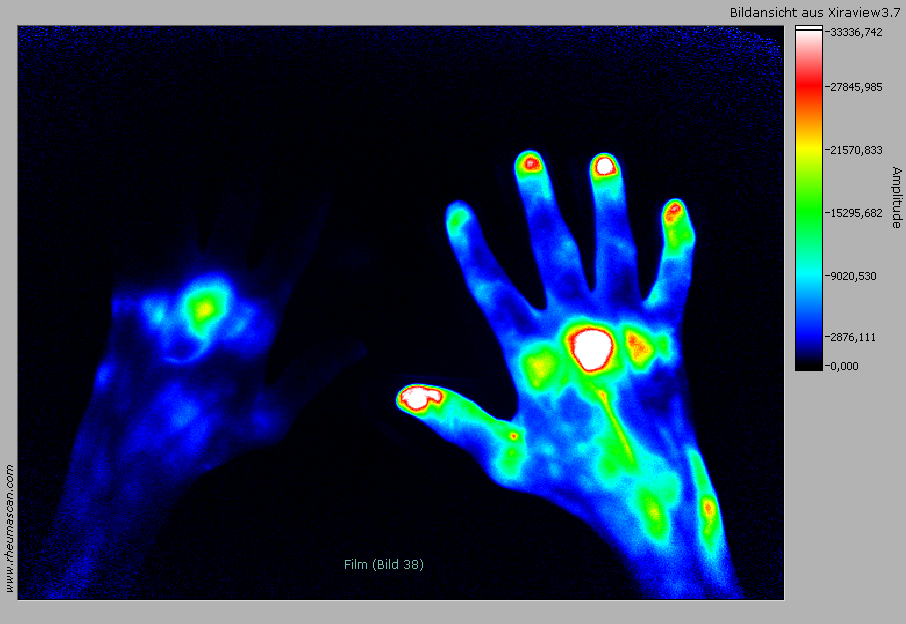

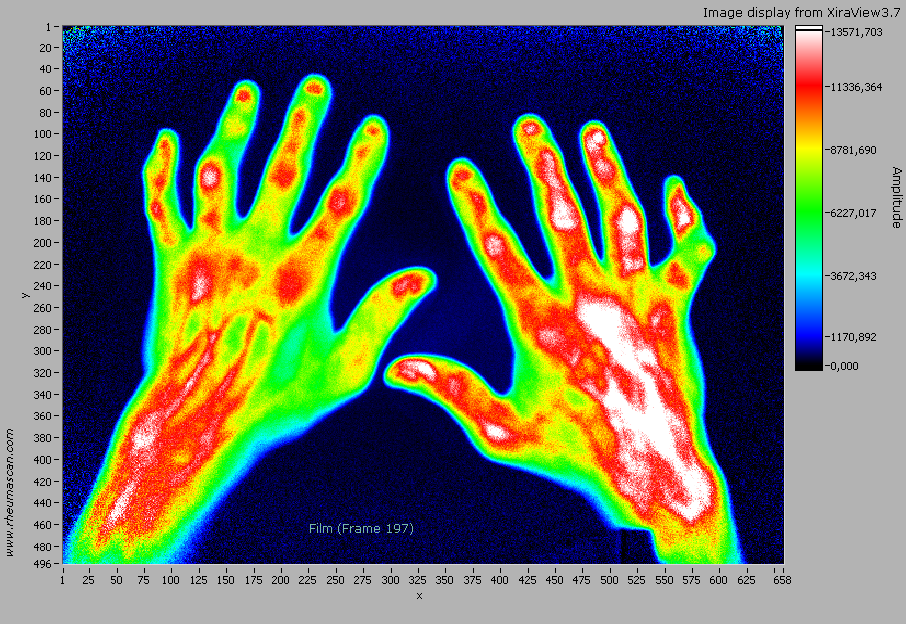


Grade 1 Grade 2 Grade 3

Wrist


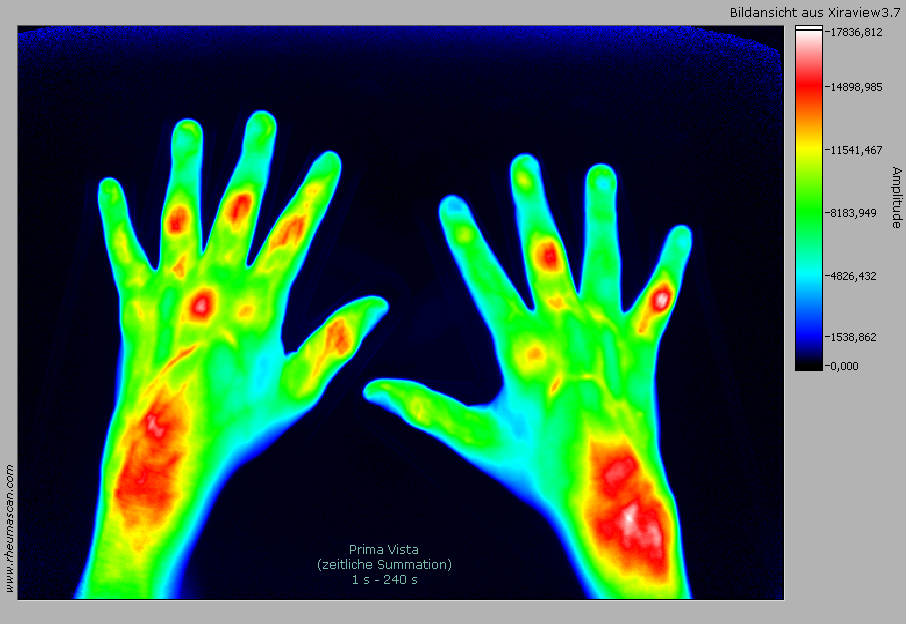

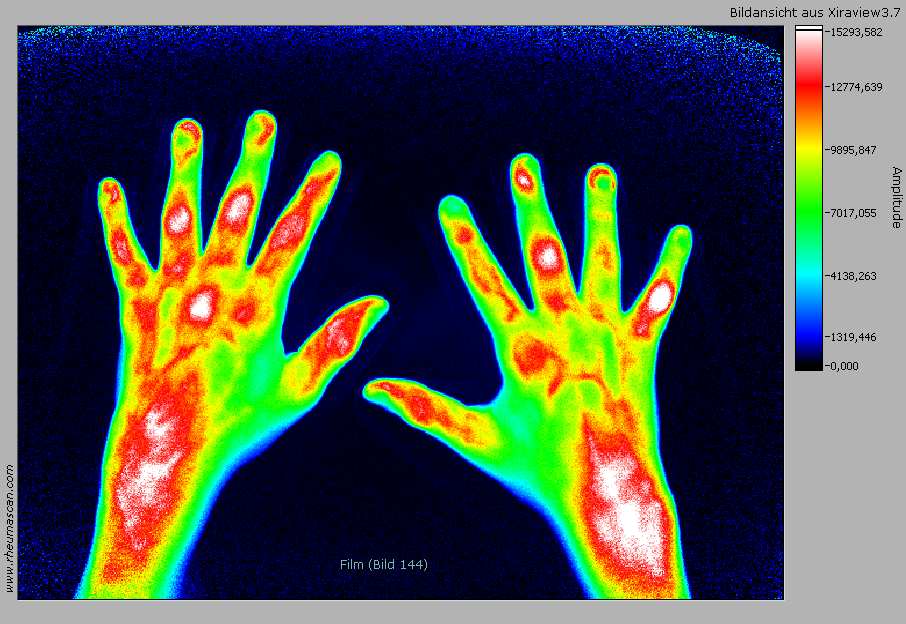

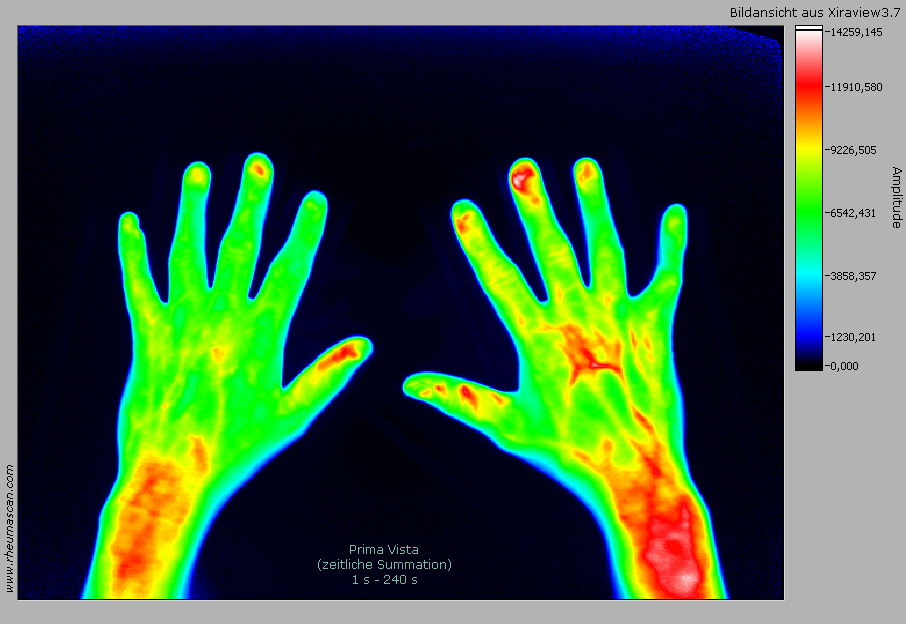


Grade 1 Grade 2 Grade 3

**Suspected Hand OA feature – «streaky signals»**

- May be a sign of chronic inflammation and degenerative disease in hand OA patients^1^
- Defined as red signal, separated by ≥ 2 stripes of yellow in the enhanced joint area. (See examples on page 13)
- Will be scored for wrist and digit 1-5.

*1. Glimm et al., Ann Rheum Dis. 2016 Mar; 75(3):566-570*

**«Streaky signals»**


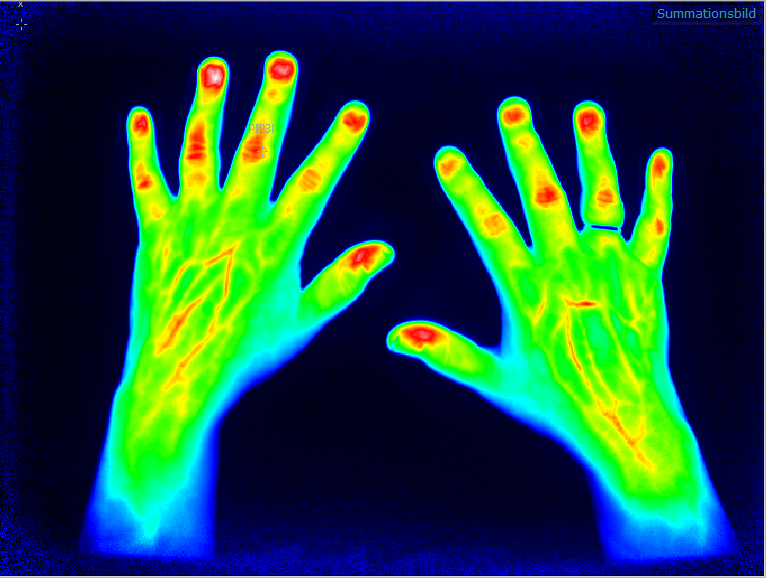

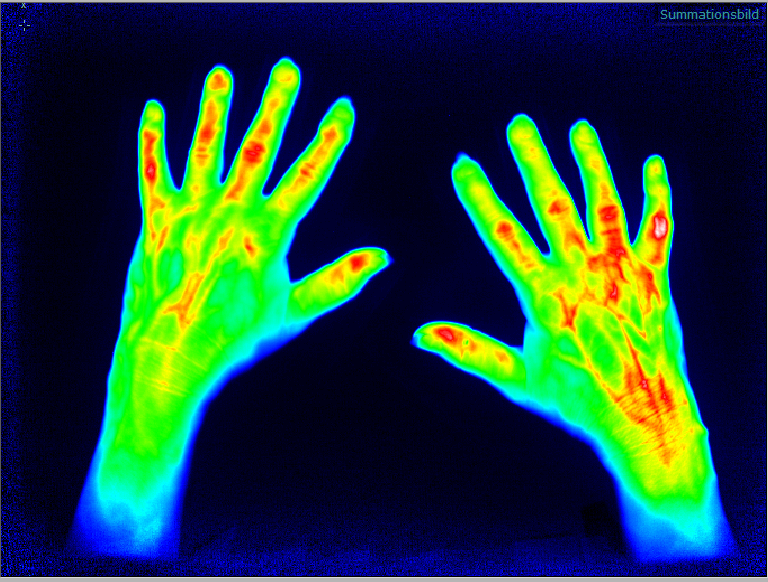


**Suspected PsA features and definitions**

- **Green nail sign**: Yellow or red signal enhancement completely surrounding a sharply demarcated green area in projection of the nail. Needs to be present during phase 2 or in PVM.^1^
- **Blue nail sign:** Extreme variant of the ‘Green nail sign’ whereas the nail appears blue.^1^
- **Werner sign:** A triangular, slightly arcuate enhancement from the nail bed into DIP joint area. Appears like a reverse pyramid. Should not be scored if the DIP joint is scored as grade ≥2 according to the FOIAS in the same finger.^2,3^
- **Bishop’s crozier sign:** Enhancement of a shape that is similar to a question mark, reverse question mark or a Bishop’s crozier next to the nail descending towards to the DIP joint.

*1 Wiemann et al. J Dtsch Dermatol Ges. 2019 Feb;17(2):138-148*

*2 Werner et al., Ann Rheum Dis. 2012 Apr; 71(4):504-510*

*3 Zeidler H 2019, Fluoreszenz optische Bildgebung. In: Zeidler H, Beat M (Hrsg.).*

*Differenzialdiagnose rheumatischer Erkrankungen 5. Aufl. Springer, Heidelberg, S. 88-89*

**
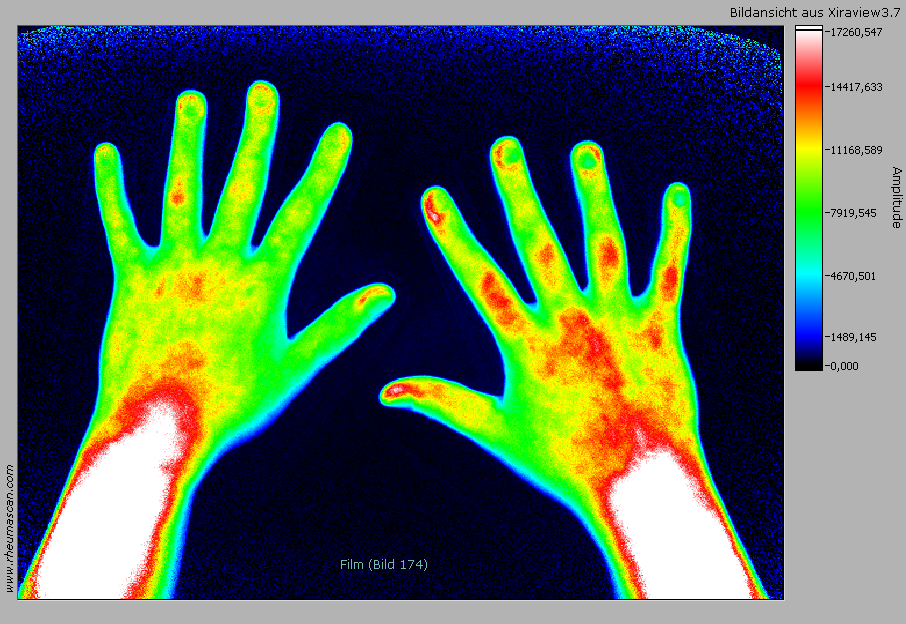

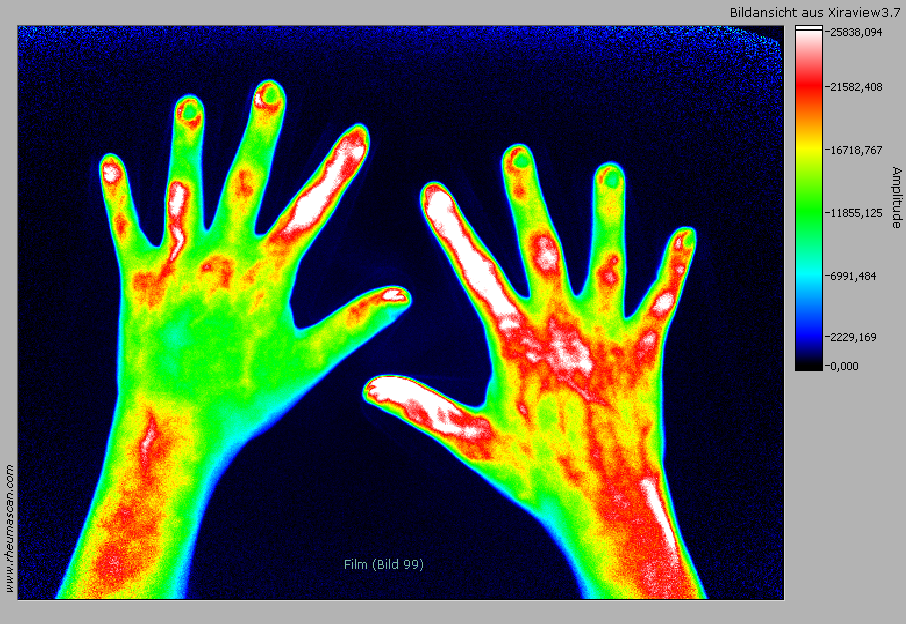

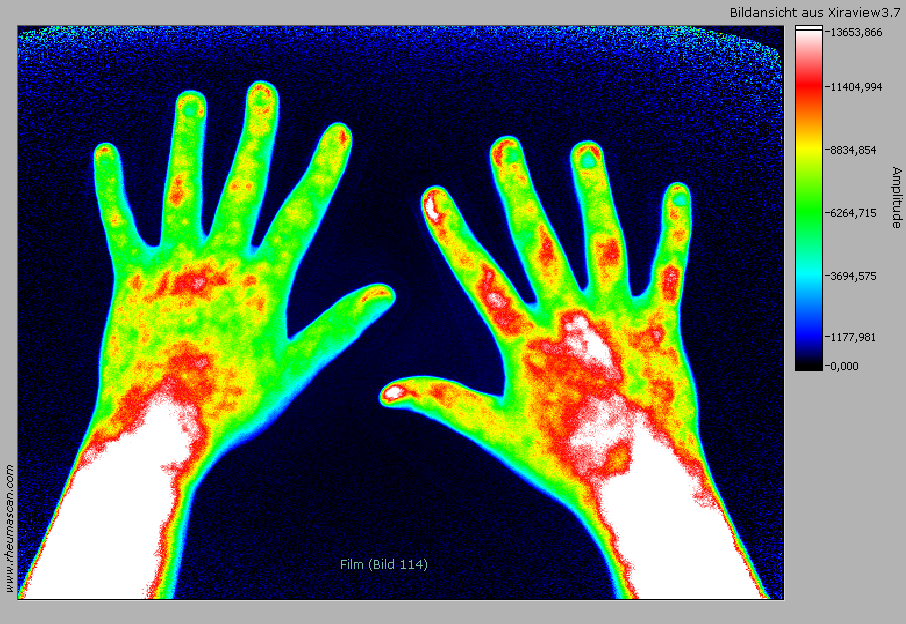
Green nail sign / Blue nail sign**

**Werner’s sign**


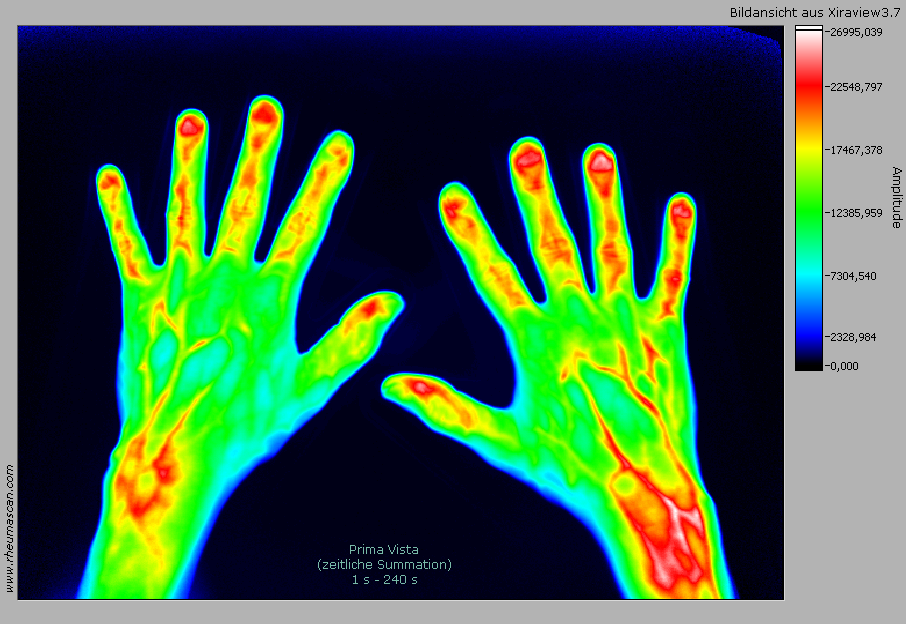


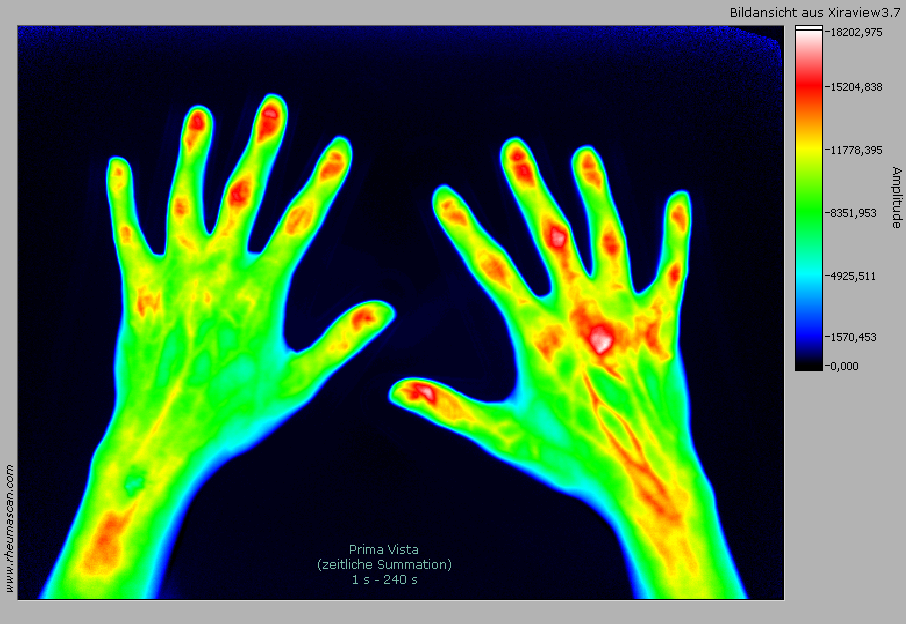

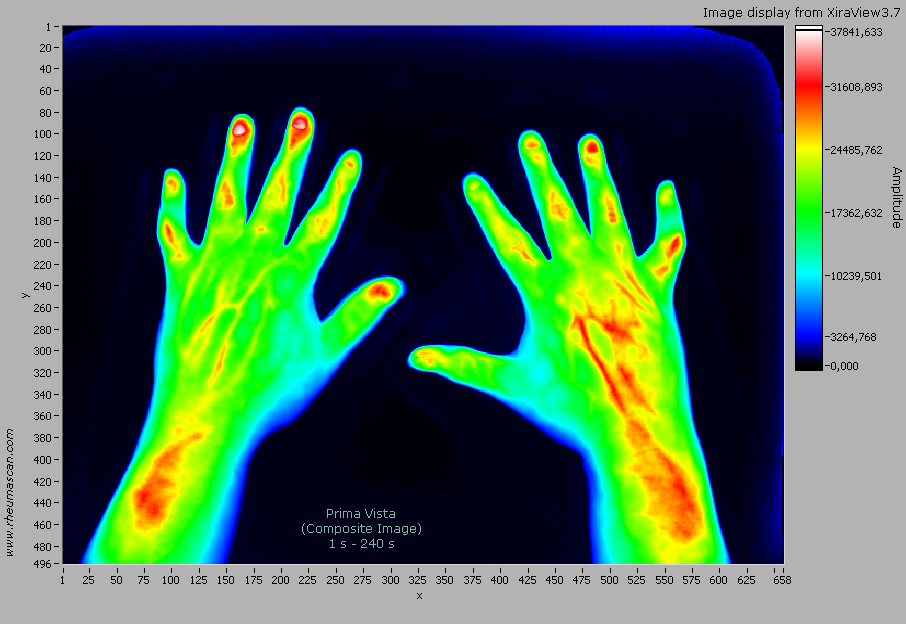


**Bishop’s crozier sign**


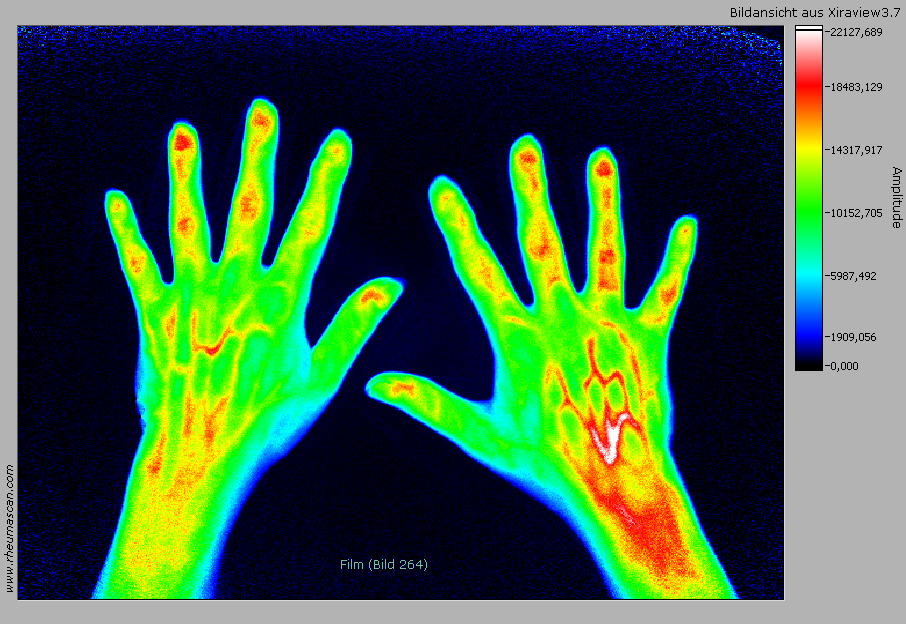

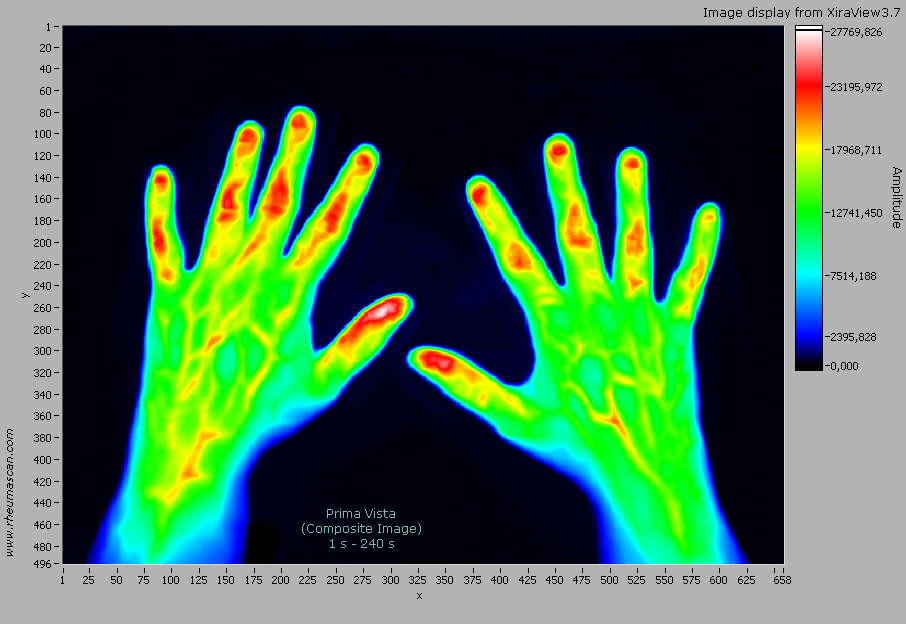


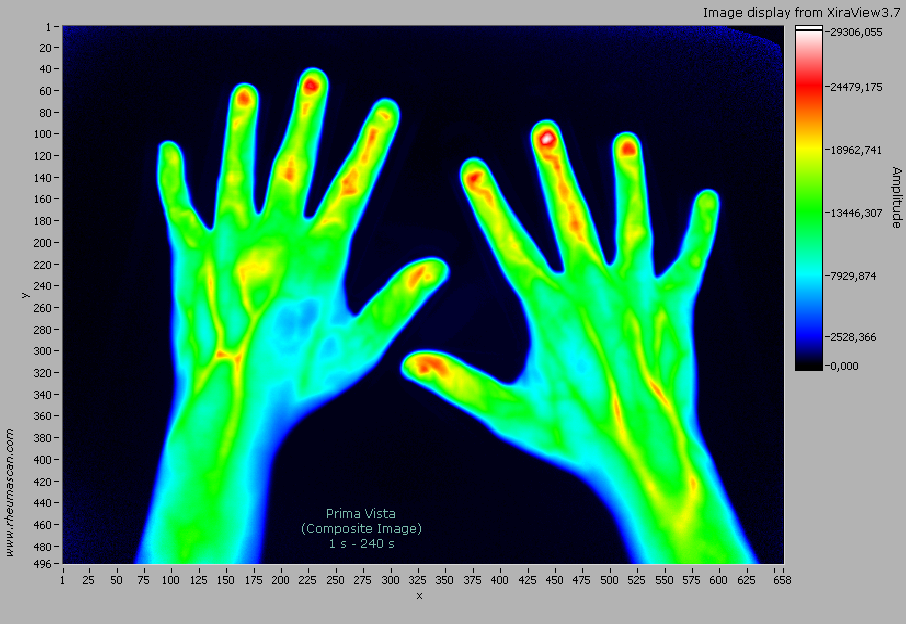

Supplement: Supplementary file 2 [file Data_Sheet_2.docx]
